# The involvement of endothelial mediators in leprosy

**DOI:** 10.1590/0074-02760160122

**Published:** 2016-10-03

**Authors:** Maria Renata Sales Nogueira, Ana Carla Pereira Latini, Maria Esther Salles Nogueira

**Affiliations:** Instituto Lauro de Souza Lima, Divisão de Pesquisa e Ensino, Secretaria de Estado da Saúde, Bauru, SP, Brasil

**Keywords:** leprosy, endothelial mediators, thrombomodulin, tissue factor

## Abstract

Leprosy is a chronic infectious disease that requires better understanding since it continues to be a significant health problem in many parts of the world. Leprosy reactions are acute inflammatory episodes regarded as the central etiology of nerve damage in the disease. The activation of endothelium is a relevant phenomenon to be investigated in leprosy reactions. The present study evaluated the expression of endothelial factors in skin lesions and serum samples of leprosy patients. Immunohistochemical analysis of skin samples and serum measurements of VCAM-1, VEGF, tissue factor and thrombomodulin were performed in 77 leprosy patients and 12 controls. We observed significant increase of VCAM-1 circulating levels in non-reactional leprosy (p = 0.0009). The immunostaining of VEGF and tissue factor was higher in endothelium of non-reactional leprosy (p = 0.02 for both) than healthy controls. Patients with type 1 reaction presented increased thrombomodulin serum levels, compared with non-reactional leprosy (p = 0.02). In type 2 reaction, no significant modifications were observed for the endothelial factors investigated. The anti-inflammatory and antimicrobial activities of the endotfhelial factors may play key-roles in the pathogenesis of leprosy and should be enrolled in studies focusing on alternative targets to improve the management of leprosy and its reactions.

Leprosy arises from infection caused by the bacteria *Mycobacterium leprae*, which produces a chronic granulomatous disease in humans that affects primarily peripheral nerves and skin (White & Franco-Paredes 2015). Once the infection is established, the occurrence of leprosy reactions constitutes the main cause of nerve dysfunction aggravation (Franco-Paredes & Rodríguez-Morales 2016). Leprosy reactions are acute inflammatory episodes superimposed to the chronic course of the disease and responsible for irreversible nerve damage (Scollard et al. 2015). Leprosy reactions are classified as type 1 (T1R) and type 2 (T2R) reactions, and show distinct immunological characteristics that may occur before and during the treatment or up to five years after the conclusion of multidrug therapy (MDT) (Pandhi & Chhabra 2013, Cortela et al. 2015).

T1R episodes are observed in borderline leprosy patients (BT, BB, BL) against antigenic determinants of *M. leprae* and epitopes exposed by immune cells during the infection (Naafs & van Hees 2016). T1R usually occurs in preexisting lesions (Scollard et al. 2006). T2R develops in lepromatous leprosy (LL) and borderline lepromatous (BL), in which is observed high bacterial load and ill-defined antigen specific T cells functions (de Souza et al. 2016). T2R is described as abruptly induced recurrent episodes of immune complex-mediated reactions, where local signs are accompanied by systemic involvement (Stefani et al. 2009, Cortela et al. 2015). The identification of specific markers of leprosy reactions could allow more efficient management of patients at higher risk. However, the risk prediction of such episodes still remains unavailable (Mizoguti et al. 2015).

Blood vessels have strategic participation on inflammation/immune response with important expression of cytokines, adhesion molecules, growth factors, and hemostatic factors by endothelial cells (ten Cate et al. 1997, Aird 2001, 2005, Levi et al. 2002, 2012). The activated endothelial cells are permeable, prothrombotic and pro-inflammatory, displaying critical roles in the sequestration and eradication of pathogens, vascular remodeling and repair (Page & Liles 2013). Circulating products from activated endothelium may be early detectable, being therefore clinically useful as predictive biomarkers in systemic infectious diseases (Page & Liles 2013).

The central role of adhesion molecules on endothelial cells involves directing the leukocyte traffic to the inflammatory sites (Alon et al. 1995). In leprosy, the elevation of adhesion molecules was demonstrated in different cell types along the spectrum of the disease. Among adhesion molecules evidenced in leprosy, it is possible to highlight the intercellular adhesion molecule-1 (ICAM-1) and its ligand, lymphocyte function-associated antigen-1 (LFA-1) (Sullivan et al. 1991, Rieckmann et al. 1996), E-selectin (Rieckmann et al. 1996), as well as vascular cell adhesion molecule 1 (VCAM-1) (Martinuzzo et al. 2002).

In granulomatous inflammation, angiogenesis upregulates the vascular endothelial growth factor (VEGF), which in turn, stimulates the migration and activation of macrophages. VEGF, first described as a product of neoplastic cells, is produced by normal somatic cells, especially of endothelial origin. VEGF increases vascular permeability and has mitogenic activity on endothelium. Increasing VEGF has been found in dermal vasculature of patients with T1R, indicating their involvement in the natural history of these episodes and potential role as a predictive marker (Fiallo et al. 2002, Soares et al. 2013, Geluk et al. 2014, Khadge et al. 2015).

The inflammation-induced expression of tissue factor (TF), also known as thromboplastin, in endothelial cells and macrophages is involved in the host immune response to infections (Liang et al. 2015). In human monocyte-derived macrophages (MDMs) infected by *Mycobacterium tuberculosis*, TF deficiency was associated with reduced expression of inducible nitric oxide synthase (iNOS), enhanced expression of arginase 1 (Arg1), elevation of IL-10 and promotion of M2-like phenotype (Venkatasubramanian et al. 2016). Furthermore, the crosstalk of TF with endothelial protein C receptor (EPCR), protease-activated receptor 2 (PAR2) and TLR4, has been suggested to be necessary for interferon-regulated host responses (Teles et al. 2013, Liang et al. 2015). Thus, the induction of TF could participate in the host response to *M. leprae*.

Thrombomodulin (TM) is a membrane protein found on the surface of the endothelial lining, where it catalyses the conversion of protein C into activated protein C (aPC) by thrombin. The transcription of TM is enhanced by arterial shear forces, involving the transcription of the Kruppel-like factor 2 (KLF-2) and the suppression of inflammatory activation (Parmar et al. 2006, van Hinsbergh 2012). TM has been described as an anti-inflammatory agent in the reduction of acute kidney injury and experimental glomerulonephritis (Ikeguchi et al. 2002, Rajashekhar et al. 2012). Additionally, diseases presenting endothelial injury including sepsis, severe malaria and dengue haemorrhagic fever display noticeable elevation of soluble TM (Boehme et al. 1994, Ohnishi 1999, Page & Liles 2013).

The activation of endothelial cells induces elevation of specific factors that may be involved in the pathogenesis of leprosy reactions. Understanding the role of endothelial mediators shall contribute to a more effective management of leprosy reactions. This study aimed to investigate the endothelial factors VCAM-1, VEGF, TM and TF in serum samples and skin lesions of leprosy patients.

## MATERIALS AND METHODS

*Subjects* - The participants of the study were selected at the outpatient clinic of Lauro de Souza Lima Institute, a reference center on leprosy, located at state of São Paulo, southeastern of Brazil. Leprosy patients were diagnosed and classified according to Ridley and Jopling (1966) criteria. At the time of sample collection, all patients were under standard MDT preconised by the World Health Organization. The moment of reaction and how long the MDT had been administered for have not been considered as exclusion criteria. Patients with concomitant systemic diseases and under corticoid therapy were excluded from the study. Participants were divided into the following groups: type 1 reaction (T1R, n = 20); type 2 reaction (T2R, n = 19); non-reactional leprosy patients (NR, n = 24) and healthy controls (HC, n = 12). Patients from NR group were subdivided into two subgroups according to their risk of developing T1R (NRT1; n = 23) and T2R (NRT2; n = 05). NRT1 has included TT, BT, BB, and BL patients, while NRT2 has included BL and LL (Table). For serum measurements, 10 mL of venous blood was obtained from participants. Serum samples were prepared and stored at -20ºC until analysis. Endothelial factors were evaluated in skin samples of T1R (n = 17), T2R (n = 13), NR (NRT1, n = 14 and NRT2, n = 03) leprosy lesions, as well as HC (n = 08).

**TABLE:** Demographic and clinical characterisation of subjects enrolled in the study

Groups	(n)	Sex (M/F)	Age^*^	Ridley and Jopling classification	BI^*^
NR	24	16/8	49 (10-79)	5TT/2BT/12BB/4BL/1LL	5 (0-6)
T1R	20	13/7	48 (17-81)	6TT/10BT/4BB/	2 (0-4)
NRT1	23	15/8	49 (10-79)	5TT/2BT/12BB/3BL	5 (0-6)
T2R	19	16/3	45(20-83)	2BL/17LL	4 (1-6)^**†**^
NRT2	5	4/1	70 (53-79)	4BL/1LL	5 (5-6)
HC	12	9/3	46 (23-69)	-	-

*: median (range); †: 8/19 (42.1%) T2R patients were diagnosed as regressive leprosy; M: male; F: female; BI: bacillary index; NR: non-reactional leprosy; T1R: type 1 reaction; NRT1: non-reactional type 1; T2R: type 2 reaction; NRT2: non-reactional type 2; HC: healthy controls; TT: tuberculoid; BT: borderline tuberculoid; BB: borderline borderline; BL: borderline lepromatous; LL: lepromatous.

*Enzyme-linked immunosorbent assay (ELISA)* - ELISA techniques were conducted according to the manufacturer’s instructions: VCAM-1 (Human VCAM-1 Quantikine® ELISA Kit, R&D Systems, USA), VEGF (Human VEGF Quantikine® ELISA Kit, R&D Systems, USA), TF (IMUBIND® Tissue Factor ELISA Kit, American Diagnostica, USA) and TM (IMUBIND® Thrombomodulin ELISA Kit, American Diagnostica, USA).

*Immunohistochemistry* - Samples were processed, embedded in paraffin and serially sectioned in the sagittal plane at 5 μM thickness in a Leica microtome. Sections were mounted on silane-coated microscope slides (ImmunoSlide, EazyPath®, Brazil). Immunohistochemical analysis was performed according to previously standardised protocol. Sections were submitted to a series of changes in xylene, ethanol and distillated water. Antigen retrieval was achieved in 10 mM sodium citrate buffer, pH 6.0, at 90ºC for 20 min, followed by incubations in TRIS-buffered saline (TBS)/0.5% Triton® X-100 and deionised water. Endogenous peroxidase activity was quenched by 3% hydrogen peroxide, for 40 min. Cross-reactivity was blocked by non-fat milk incubation, for 30 min. Microscopic sections were then incubated overnight with primary mouse/goat anti-human antibodies to VCAM-1 (Santa Cruz Biotechnology Inc., USA), VEGF (R&D Systems, USA), TM (Santa Cruz Biotechnology Inc., USA) and TF (R&D Systems, USA). Subsequently, samples were incubated in TBS/0.5% Triton® X-100, biotinylated secondary antibodies and streptavidin-conjugated HRP (LSAB^+^System-HRP Universal, DakoCytomation, Denmark), for 30 min each. Substrate chromogen solution (DAB - 3,3’-diaminobenzidine, DakoCytomation, Denmark) was applied on sections and blocked after 1-5 min. Further, the sections were counter-stained with Harris Hematoxylin for 3 min, mounted in Permount™ Mounting Medium (Fischer Scientific, USA) and visualised by standard light microscope. Immunohistochemical staining was assessed in blood vessels located in granulomatous lesions of leprosy patients (T1R, T2R, NRT1, and NRT2) and in blood vessels of normal dermis in HC group. All sections were evaluated under 400x magnification. Semi-quantitative scores for each area were assigned as follows: (0) negative; (1) weak positivity; (2) positive. It was not necessary to perform a blind analysis on immunostained sections since the evaluation criteria were based on the above-mentioned predefined scores and specific microscopic fields.

*Statistical analysis* - Results were expressed by mean ± standard deviation. The data from serum measurements were submitted to the unpaired t test or non-parametric Mann-Whitney test, depending on the results of Shapiro-Wilk normality test. The data collected from immunohistochemical analysis were submitted to non-parametric Mann-Whitney test. GraphPad Prism 5.01 for Windows (GraphPad Software Inc., USA) was used for statistical analysis. P value < 0.05 was considered as cutoff for significance.

*Ethics* - Procedures were in accordance with the ethical standards of the Human Ethics Committee of the Lauro de Souza Lima Institute and the Helsinki Declaration. All participants were informed about the study aims and the procedures involved, then included only after signing the Informed Consent Form, in accordance with Resolution nº 466 (2012) of the National Health Council.

## RESULTS

It was observed herein that circulating levels of the majority of endothelial factors are elevated in leprosy patients and that a marked TM increase was verified in T1R as well. The population of study was composed by 67 leprosy patients and 12 healthy controls, detailed in the Table.

**Fig. 1:**
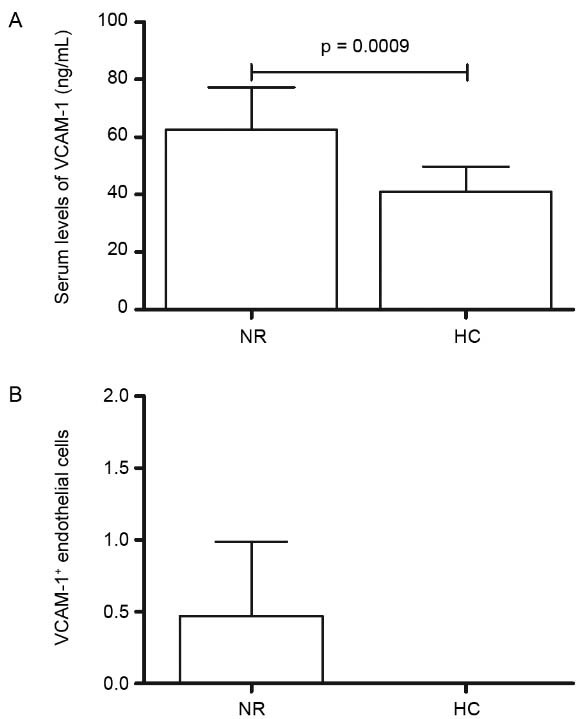
: VCAM-1 in leprosy. (A) Comparison of VCAM-1 serum levels in non-reactional leprosy patients (NR) *vs* healthy controls (HC) (p = 0.0009); (B) immunohistochemical findings of VCAM-1 in skin lesions of NR *vs* HC. Unpaired t test and Mann-Whitney test were applied. P value < 0.5 was taken as significance level.

ELISA evaluation of VCAM-1 in NR leprosy (62.5 ± 14.7) demonstrated higher levels than HC (40.9 ± 8.9) (p = 0.0009) (Fig. 1A). However, T1R patients (67.7 ± 28.1) and T2R (57.9 ± 17.1) did not present distinct levels compared to NRT1 (60.8 ± 14.5) and NRT2 (75.29 ± 8.56). Immunohistochemical results for VCAM-1 in T1R and T2R compared to NRT1 and NRT2 have also showed no substantial differences. VCAM-1 was not detectable in HC tissue samples (Fig. 1B).

**Fig. 2:**
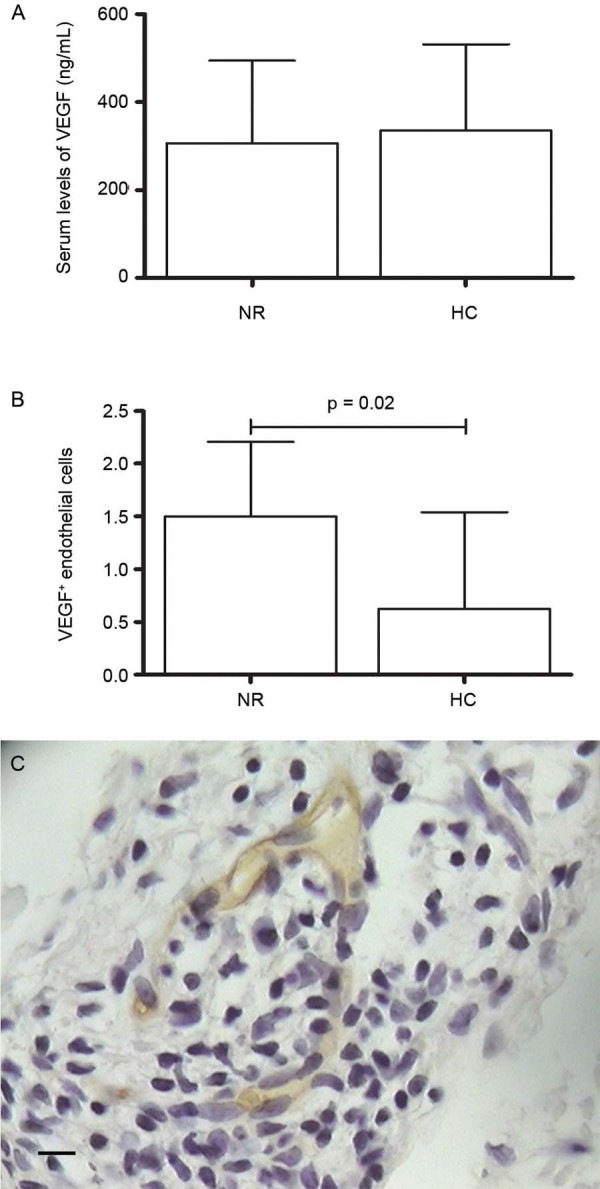
: VEGF in leprosy. (A) Circulating levels of VEGF in non-reactional leprosy patients (NR) *vs* healthy controls (HC); (B) immunohistochemical evaluation of VEGF in NR lesions compared to HC (p = 0.02); (C) VEGF-positive endothelial cells in NR leprosy lesions (400x magnification; Bar: 25 µM). Mann-Whitney test was applied. P value < 0.5 was taken as significance level.

Serum levels of VEGF were not different between NR (306.9 ± 187.6) and HC (336.0 ± 195.5) (Fig. 2A); the same has been observed when we compared T1R (333.4 ± 74.3) with NRT1 (267.5 ± 148.9) or T2R (515.4 ± 313.2) and NRT2 samples (444.5 ± 189.7). However, immunohistochemical evaluation in NR leprosy lesions demonstrated more VEGF-positive endothelial cells than in HC (p = 0.02) (Fig. 2B-C).

**Fig. 3:**
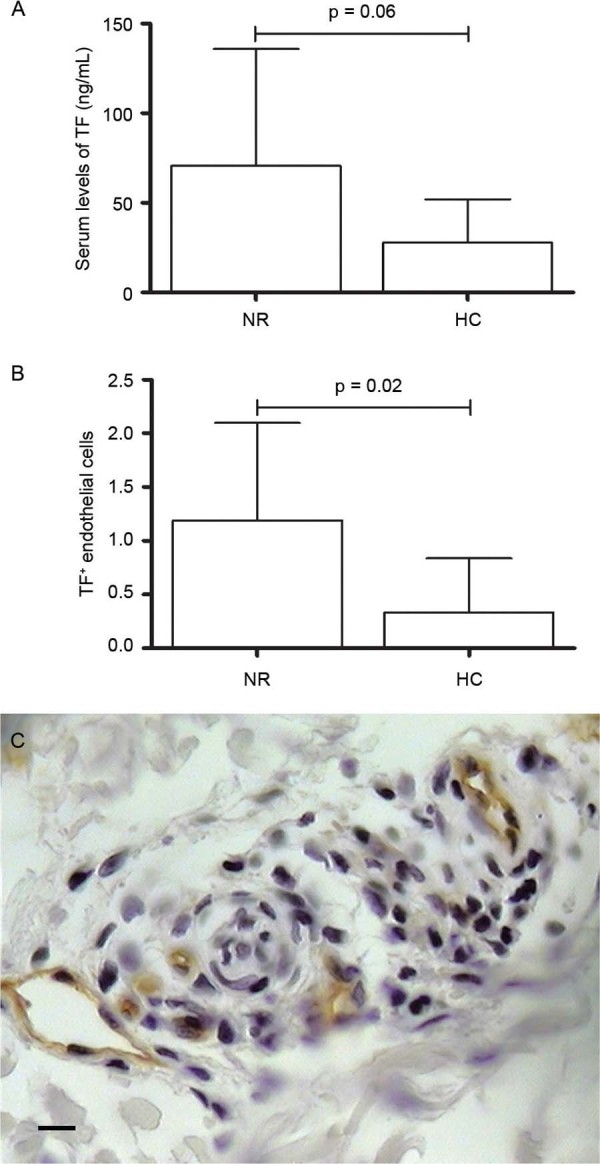
: tissue factor (TF) in leprosy. (A) Serum levels of TF in non-reactional leprosy (NR) showing a trend to statistical significance when compared to healthy controls (HC) (p = 0.06); (B) immunohistochemical data of TF in NR *vs* HC (p = 0.02); (C) TF immunostaining in endothelium of NR leprosy patients (400x magnification; Bar: 25 µM). Mann-Whitney test was applied. P value < 0.5 was taken as significance level.

Circulating levels of TF in NR patients (70.5 ± 65.4) compared to HC (27.7 ± 24.1) revealed a trend towards statistically significant increase (p = 0.06) (Fig. 3A). TF levels in T1R (68.4 ± 38.6) and T2R (84.8 ± 103.6) were not different from NRT1 (41.4 ± 26.3) and NRT2 (136.5 ± 74.8), respectively. Immunohistochemical analysis followed serum results and TF was statistically different only when we compared NR *versus* HC (p = 0.02) (Fig. 3B-C).

**Fig. 4:**
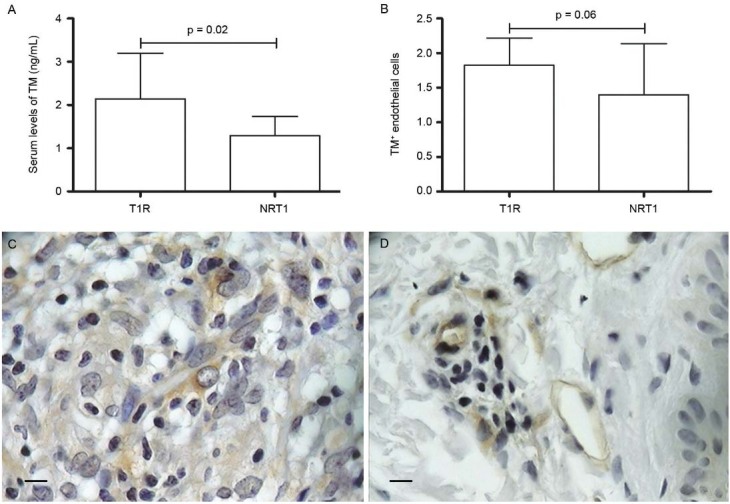
: thrombomodulin (TM) in type 1 leprosy reaction. (A) Serum levels of TM in type 1 reaction (T1R) compared to non-reactional leprosy (NRT1) (p = 0.02). (B) immunohistochemical findings of TM in T1R *vs* NRT1 (p = 0.06); (C-D) TM staining in T1R and NRT1 (400x magnification; Bar: 25 µM). Mann-Whitney test was applied. P value < 0.5 was taken as significance level.

Soluble form of TM was significantly elevated (p = 0.02) in T1R (2.14 ± 1.05) compared to NRT1 (1.29 ± 0.44) (Fig. 4A). TM serum levels in NR patients (0.62 ± 1.90) were not distinct from HC (0.06 ± 2.15), as well as TM in T2R (1.57 ± 0.77) compared to NRT2 (1.66 ± 0.01). Immunohistochemical expression of TM in endothelial cells followed the ELISA results, demonstrating a trend to be higher (p = 0.06) in T1R compared to NRT1 (Fig. 4B-D).

## DISCUSSION

Endothelial activation produces a variety of molecules that play key roles in the pathogenesis of leprosy and leprosy reactions.

TM is recognised for its important anti-inflammatory role on the protection of endothelium. It has been shown that the treatment with soluble forms of TM (sTM) reduced the expression of adhesion molecules in endothelial cells activated by TNF (Rajashekhar et al. 2012). The mechanisms by which TM exerts its anti-inflammatory effect also involve complement and NF-kappa B pathways inhibition (Conway et al. 2002). TMD1 is a C-type lectin domain at the TM molecule responsible for its anti-inflammatory effect. Interestingly, TMD1 domain is similar to those present at the C-type lectin receptors MRC1 and MBL, which in leprosy are widely involved in the innate immunity against *M. leprae* (Cardoso et al. 2011).

Li et al. (2012) have demonstrated an anti-angiogenic effect of the recombinant TMD1 by interacting with Lewis Y antigen (LeY). Noticeably, leprosy reactions may be locally characterised by the induction of angiogenesis, evidenced by CD31^+^ and CD105^+^ cells (Soares et al. 2013). Soares et al. (2013) have verified an inverse correlation between angiogenesis and the regression of leprosy lesions, suggesting that the control of local angiogenesis could provide a strategy to limit damage caused by leprosy reactions. We hypothesize that the TM increase in T1R could be a protection mechanism to contain angiogenesis and the exacerbation of inflammation.

Toda et al. (2013) have demonstrated two subsets of dendritic cells (DCs) classified according to TM expression. TM^**+**^ DCs presented a tolerogenic profile, while TM^-^ DCs revealed immunogenic behavior. The expression of TM in DCs decreases the maturation markers and inflammatory cytokines as TNF, IL-12p70 and IL-6, while increases IL-10 production. Furthermore, T1R presents higher number of DCs than T2R (Miranda et al. 2007). Since distinct immune mechanisms characterise T1R and T2R development, it is feasible that TM is induced in leprosy reactions by a range of influences. In line with such hypothesis, elevated levels of COX-2 in T1R may contribute to the production of TM, throughout PGI2 (Rabausch et al. 2005, Pesce et al. 2006). Our results reinforce previous statements on sTM as a promising alternative for non-steroidal and non-arachidonic acid anti-inflammatory agents (Rajashekhar et al. 2012).

TF is a key mediator of the coagulation pathway (van Hinsbergh 2012). In addition to its prothrombotic activities, TF elevation is associated to TLR3-dependent interferon beta (IFN-β) expression (Bode & Mackman 2015, Liang et al. 2015) that suppresses IFN-γ-induced antimicrobial responses in human leprosy (Teles et al. 2013, 2015). The positive correlation observed between serum levels of TF and bacillary index (r = 0.65, p = 0.05), as well as a trend towards the increase of TF serum levels in NR leprosy patients compared to HC could be associated with the host failure attempting to control the infection. ELISA results were reinforced by TF immunostaining in leprosy lesions, in which TF-positive blood vessels of NR were significantly higher than HC tissue samples. Conversely, in vitro experiments and knockout animal model data have revealed that TF deficiency contributes to the growth of *M. tuberculosis* by mechanisms involving the induction of M2-like phenotype (Venkatasubramanian et al. 2016). Forastiero et al. (2005) have not found high levels of TF in leprosy patients compared to healthy controls. These distinct findings could be attributed to experimental design disparities.

The interaction between the adhesion molecules VLA-4 and VCAM-1 is early involved in transendothelial migration of T lymphocytes and its retention into the tissues (van Dinther-Janssen et al. 1991, Wang et al. 2014). A range of inflammatory mediators, as TNF, IL-6 and TGF-β1 is able to increase VCAM-1 on activated endothelial cells (Pierangeli et al. 1999, Kaplanski et al. 2000, Wang et al. 2014). In spite of the clear significance of VCAM-1 in inflammation, only a previous study has shown the elevation of VCAM-1 in non-reactional leprosy patients with antiphospholipid antibodies (Martinuzzo et al. 2002). Herein, we demonstrate a noticeable increase of VCAM-1 in NR leprosy patients compared to HC. However, we observed that T2R presented lower serum titles of VCAM-1 in comparison to NRT2 and NR samples, maybe due to the widely variable VCAM-1 production in T2R, and the relative small number of NRT2 patients enrolled in our study. Additionally, the majority of T2R patients were LL (89.4%), while the NRT2 patients were BL (80%). We could not reject the possible influence of clinical forms of leprosy in final results of VCAM-1. Similar findings were observed in TF evaluation, probably for the same reasons argued in the case of VCAM-1. The distribution of clinical forms in T2R and NRT2 did not influence VEGF and TM results. Previous studies have demonstrated that ICAM-1 in keratinocytes (Sullivan et al. 1991) and E-selectin (Souza et al. 2015) in endothelial cells could be correlated with the outcome of the host response to infection. We suggest a comparable role of VCAM-1 with the former adhesion molecules, in the endothelium of leprosy patients.

The overexpression of VEGF was previously associated to severity of infectious conditions in which the elevation of soluble TM and VCAM-1 was identified (Page & Liles 2013). In leprosy, the overexpression of VEGF was reported in T1R patients (Fiallo et al. 2002, Soares et al. 2013, Geluk et al. 2014, Khadge et al. 2015). Although VEGF is very likely involved in T1R, our data demonstrated a significant increase of VEGF only in NR skin lesions compared to HC.

The importance of interplay between endothelial cells with *M. leprae* and the limited data on this issue should be addressed in further studies. We have evaluated endothelial factors in human umbilical endothelial cell (HUVEC) culture stimulated by inactivated *M. leprae*, after 24 h (data not shown). No significant differences were observed. Maybe alternative experimental conditions could provide more clarifying results.

The knowledge about leprosy reactions has led to a better understanding of immune mechanisms and efforts in the discovery of biomarkers. Notwithstanding, still remains a limited scenario of options in the prediction and treatment of these inflammatory episodes. Our study discusses the roles of endothelial mediators in the pathogenesis of leprosy and their reactions in order to contribute to studies focusing on alternative targets to improve the management of leprosy.

## References

[B1] Aird WC (2005). Spatial and temporal dynamics of the endothelium. J Thromb Haemost.

[B2] Aird WC (2001). Vascular bed-specific hemostasis: role of endothelium in sepsis
pathogenesis. Crit Care Med.

[B3] Alon R, Kassner PD, Carr MW, Finger EB, Hemler ME, Springer TA (1995). The integrin VLA-4 supports tethering and rolling in flow on
VCAM-1. J Cell Biol.

[B4] Bode MF, Mackman N (2015). Protective and pathological roles of tissue factor in the
heart. Hamostaseologie.

[B5] Boehme MW, Nawroth PP, Kling E, Lin J, Amiral J, Riedesel J (1994). Serum thrombomodulin. A novel marker of disease activity in systemic
lupus erythematosus. Arthritis Rheum.

[B6] Cardoso CC, Pereira AC, Marques CS, Moraes MO (2011). Leprosy susceptibility: genetic variations regulate innate and
adaptive immunity, and disease outcome. Future Microbiol.

[B7] Conway EM, Van de Wouwer M, Pollefeyt S, Jurk K, Van Aken H, De Vriese A (2002). The lectin-like domain of thrombomodulin confers protection from
neutrophil-mediated tissue damage by suppressing adhesion molecule expression via
nuclear factor κB and mitogen-activated protein kinase pathways. J Exp Med.

[B8] Cortela DC, Souza AL de, Virmond MC, Ignotti E (2015). Inflammatory mediators of leprosy reactional episodes and dental
infections: a systematic review. Mediators Inflamm.

[B9] Souza VN, Iyer AM, Lammas DA, Naafs B, Das PK (2016). Advances in leprosy immunology and the field application: a gap to
bridge. Clin Dermatol.

[B10] Fiallo P, Clapasson A, Favre A, Pesce C (2002). Overexpression of vascular endothelial growth factor and its
endothelial cell receptor KDR in type 1 leprosy reaction. Am J Trop Med Hyg.

[B11] Forastiero RR, Martinuzzo ME, Larrañaga GF (2005). Circulating levels of tissue factor and proinflammatory cytokines in
patients with primary antiphospholipid syndrome or leprosy related
antiphospholipid antibodies. Lupus.

[B12] Franco-Paredes C, Rodríguez-Morales AJ (2016). Unsolved matters in leprosy: a descriptive review and call for further
research. Ann Clin Microbiol Antimicrob.

[B13] Geluk A, van Meijgaarden KE, Wilson L, Bobosha K, van der Ploeg-van Schip JJ, van den Eeden SJ (2014). Longitudinal immune responses and gene expression profiles in type 1
leprosy reactions. J Clin Immunol.

[B14] Ikeguchi H, Maruyama S, Morita Y, Fujita Y, Kato T, Natori Y (2002). Effects of human soluble thrombomodulin on experimental
glomerulonephritis. Kidney Int.

[B15] Kaplanski G, Cacoub P, Farnarier C, Marin V, Grégoire R, Gatel A (2000). Increased soluble vascular cell adhesion molecule-1 concentrations in
patients with primary or systemic lupus erythematosus-related antiphospholipid
syndrome: correlations with the severity of thrombosis. Arthritis Rheum.

[B16] Khadge S, Banu S, Bobosha K, van der Ploeg-van Schip JJ, Goulart IM, Thapa P (2015). Longitudinal immune profiles in type 1 leprosy reactions in
Bangladesh, Brazil, Ethiopia and Nepal. BMC Infect Dis.

[B17] Levi M, ten Cate H, van der Poll T (2002). Endothelium: interface between coagulation and
inflammation. Crit Care Med.

[B18] Levi M, van der Poll T, Schultz M (2012). New insights into pathways that determine the link between infection
and thrombosis. Neth J Med.

[B19] Li YH, Kuo CH, Shi GY, Wu HL (2012). The role of thrombomodulin lectin-like domain in
inflammation. J Biomed Sci.

[B20] Liang HP, Kerschen EJ, Hernández I, Basu S, Zogg M, Botros F (2015). EPCR-dependent PAR2 activation by the blood coagulation initiation
complex regulates LPS-triggered interferon responses in mice. Blood.

[B21] Martinuzzo ME, Larranaga GF, Forastiero RR, Pelegri Y, Fariña MH, Alonso BS (2002). Markers of platelet, endothelial cell and blood coagulation activation
in leprosy patients with antiphospholipid antibodies. Clin Exp Rheumatol.

[B22] Miranda A, Amadeu TP, Schueler G, Alvarenga FB, Duppré N, Ferreira H (2007). Increased Langerhans cell accumulation after mycobacterial
stimuli. Histopathology.

[B23] Mizoguti DF, Hungria EM, Freitas AA, Oliveira RM, Cardoso LPV, Costa MB (2015). Multibacillary leprosy patients with high and persistent serum
antibodies to leprosy IDRI diagnostic-1/LID-1: higher susceptibility to develop
type 2 reactions. Mem Inst Oswaldo Cruz.

[B24] Naafs B, van Hees CL (2016). Leprosy type 1 reaction (formerly reversal reaction). Clin Dermatol.

[B25] Ohnishi K (1999). Serum levels of thrombomodulin, intercellular adhesion molecule-1,
vascular cell adhesion molecule-1 and E-selectin in the acute phase of Plasmodium
vivax malaria. Am J Trop Med Hyg.

[B26] Page AV, Liles WC (2013). Biomarkers of endothelial activation/dysfunction in infectious
diseases. Virulence.

[B27] Pandhi D, Chhabra N (2013). New insights in the pathogenesis of type 1 and type 2 lepra
reaction. Indian J Dermatol Venereol Leprol.

[B28] Parmar KM, Larman HB, Dai GH, Zhang YH, Wang ET, Moorthy SN (2006). Integration of flow dependent endothelial phenotypes by Kruppel-like
factor 2. J Clin Investig.

[B29] Pesce C, Grattarola M, Menini S, Fiallo P (2006). Cyclooxygenase 2 expression in vessels and nerves in reversal reaction
leprosy. Am J Trop Med Hyg.

[B30] Pierangeli SS, Colden-Stanfield M, Liu X, Barker JH, Anderson GL, Harris EN (1999). Antiphospholipid antibodies from antiphospholipid syndrome patients
activate endothelial cells in vitro and in vivo. Circulation.

[B31] Rabausch K, Bretschneider E, Sarbia M, Meyer-Kirchrath J, Censarek P, Pape R (2005). Regulation of thrombomodulin expression in human vascular smooth
muscle cells by COX-2-derived prostaglandins. Circ Res.

[B32] Rajashekhar G, Gupta A, Marin A, Friedrich J, Willuweit A, Berg DT (2012). Soluble thrombomodulin reduces inflammation and prevents
microalbuminuria induced by chronic endothelial activation in transgenic
mice. Am J Physiol Renal Physiol.

[B33] Ridley DS, Jopling WH (1966). Classification of leprosy according to immunity. A five-group
system. Int J Lepr Other Mycobact Dis.

[B34] Rieckmann P, Scholze G, Weichselbraun I, Ganapati R, Prange HW (1996). Soluble adhesion molecules in sera of patients with leprosy: levels of
soluble intercellular adhesion molecule-1 (sICAM-1) rapidly decrease during
multi-drug therapy. Clin Exp Immunol.

[B35] Scollard DM, Adams LB, Gillis TP, Krahenbuhl JL, Truman RW, Williams DL (2006). The continuing challenges of leprosy. Clin Microbiol Rev.

[B36] Scollard DM, Martelli CM, Stefani MM, Maroja MF, Villahermosa L, Pardillo F (2015). Risk factors for leprosy reactions in three endemic
countries. Am J Trop Med Hyg.

[B37] Soares CT, Rosa PS, Trombone AP, Fachin LR, Ghidella CC, Ura S (2013). Angiogenesis and lymphangiogenesis in the spectrum of leprosy and its
reactional forms. PLoS ONE.

[B38] Souza J, Sousa JR, Hirai KE, Silva LM, Fuzii HT, Dias LB (2015). E-selectin and P-selectin expression in endothelium of leprosy skin
lesions. Acta Trop.

[B39] Stefani MM, Guerra JG, Sousa AL, Costa MB, Oliveira ML, Martelli CT (2009). Potential plasma markers of Type 1 and Type 2 leprosy reactions: a
preliminary report. BMC Infect Dis.

[B40] Sullivan L, Sano S, Pirmez C, Salgame P, Müeller C, Hofman F (1991). Expression of adhesion molecules in leprosy lesions. Infect Immun.

[B41] Teles RM, Graeber TG, Krutzik SR, Montoya D, Schenk M, Lee DJ (2013). Type I interferon suppresses type II interferon-triggered human
anti-mycobacterial responses. Science.

[B42] Teles RM, Kelly-Scumpia KM, Sarno EN, Rea TH, Ochoa MT, Cheng G (2015). IL-27 Suppresses sntimicrobial activity in human
leprosy. J Invest Dermatol.

[B43] ten Cate JW, van der Poll T, Levi M, ten Cate H, van Deventer SJ (1997). Cytokines: triggers of clinical thrombotic disease. Thromb Haemost.

[B44] Toda M, Shao Z, Yamaguchi KD, Takagi T, D’Alessandro-Gabazza CN, Taguchi O (2013). Differential gene expression in thrombomodulin (TM; CD141)(+) and
TM(-) dendritic cell subsets. PLoS ONE.

[B45] van Dinther-Janssen AC, Horst E, Koopman G, Newmann W, Scheper RJ, Meijer CJ (1991). The VLA-4/VCAM-1 pathway is involved in lymphocyte adhesion to
endothelium in rheumatoid synovium. J Immunol.

[B46] van Hinsbergh VW (2012). Endothelium - role in regulation of coagulation and
inflammation. Semin Immunopathol.

[B47] Venkatasubramanian S, Tripathi D, Tucker T, Paidipally P, Cheekatla S, Welch E (2016). Tissue factor expression by myeloid cells contributes to protective
immune response against Mycobacterium tuberculosis infection. Eur J Immunol.

[B48] Wang PC, Weng CC, Hou YS, Jian SF, Fang KT, Hou MF (2014). Activation of VCAM-1 and its associated molecule CD44 leads to
increased malignant potential of breast cancer cells. Int J Mol Sci.

